# Low dose corticosteroid in association with methotrexate for therapy of ocular sarcoidosis: report of a case

**DOI:** 10.1186/s40942-015-0006-7

**Published:** 2015-06-26

**Authors:** Grasiane Nunes Mayer, Morgana Longo, Bárbara Brandi Gomes, Mário Junqueira Nóbrega

**Affiliations:** 1 Resident in Ophthalmology, Sadalla Amin Ghanem Eye Hospital, Rua Desembargador Nelson Nunes Guimarães, 811, CEP 89203060 Joinville, SC Brasil; 2 University of Joinville, Rua Saí, 305, apto 704, Anita Garibaldi, CEP 89202-170 Joinville, SC Brazil; 3 University of Joinville, Rua Alexandre Schlemm, 233, apto 301, Bucarein, CEP 89202-417 Joinville, SC Brazil; 4 University of Joinville, Sadalla Amin Ghanem Eye Hospital, Rua Camboriú, 35 (Eixo Marquês de Olinda), CEP 89216-222 Joinville, SC Brazil

**Keywords:** Ocular sarcoidosis, Sarcoidosis in Ophthalmology, Diagnosis of sarcoidosis

## Abstract

Sarcoidosis is a multisystem granulomatous disease of unknown etiology. Ocular involvement can be the initial manifestation, occurring by itself, or it can be associated with other systemic signs of sarcoidosis. A 31 years-old caucasian female presented a 10-day history of decreased vision and pain in OS. Biomicroscopy revealed fine keratic precipitates, a mild reaction in the anterior chamber and in the vitreous cavity in both eyes and a small posterior synechiae in OS. Intraocular pressure was 12 mmHg in the OD and 9 mmHg in OS. Fundoscopy disclosed mild swollen and hyperemic optic discs and some subretinal yellowish nodules of 1/3 to 1 disc diameter, partially delimited, located in the posterior pole and midperiphery in both eyes. The Chest X-ray disclosed the suspicion of hilar lymphadenopathy, especially on the left-side. The positron emission tomography (PET-scan) showed increased 18 F-fluorodeoxyglucose uptake in the paratracheal and bilateral hilar lymph nodes, indicating high local cellular metabolism. A mediastinal lymph node biopsy was performed afterwards, which depicted non-caseating granulomas with multinucleated giant cells and absence of acid-alcohol resistant bacilli. The patient was initially treated with oral prednisone 60 mg/day with slow tapering over the next months. A decreased papillitis and an increased size of the choroidal granulomas was observed after a 6-month therapy (Figs. 1B and 2B). Then, a combination of methotrexate 20 mg/week and prednisone 10 mg/day was given over the following months, which led to a gradual reduction in size and thickness of the choroidal granulomas. A weak response to steroid monotherapy was initially observed in this patient. The introduction of a non-steroid immunosuppressant drug (methotrexate) was associated with significant anatomic improvement in the following months. This report raises the suggestion that an early introduction of a nonsteroidal immunosuppressive drug to systemic steroids may be beneficial for a rapid remission of the uveitis.

## Background

Sarcoidosis is a multisystem granulomatous disease of unknown etiology [1]. Ocular involvement can be the initial manifestation, occurring by itself, or it can be associated with other systemic signs of sarcoidosis [2]; eyes are affected in 25 to 50 % of patients [3, 4]. Early and effective treatment of ocular sarcoidosis is critical because of the high risk of progression to irreversible vision loss, especially for glaucoma and cystoid macular edema [5].

## Case presentation

**Fig. 1:**
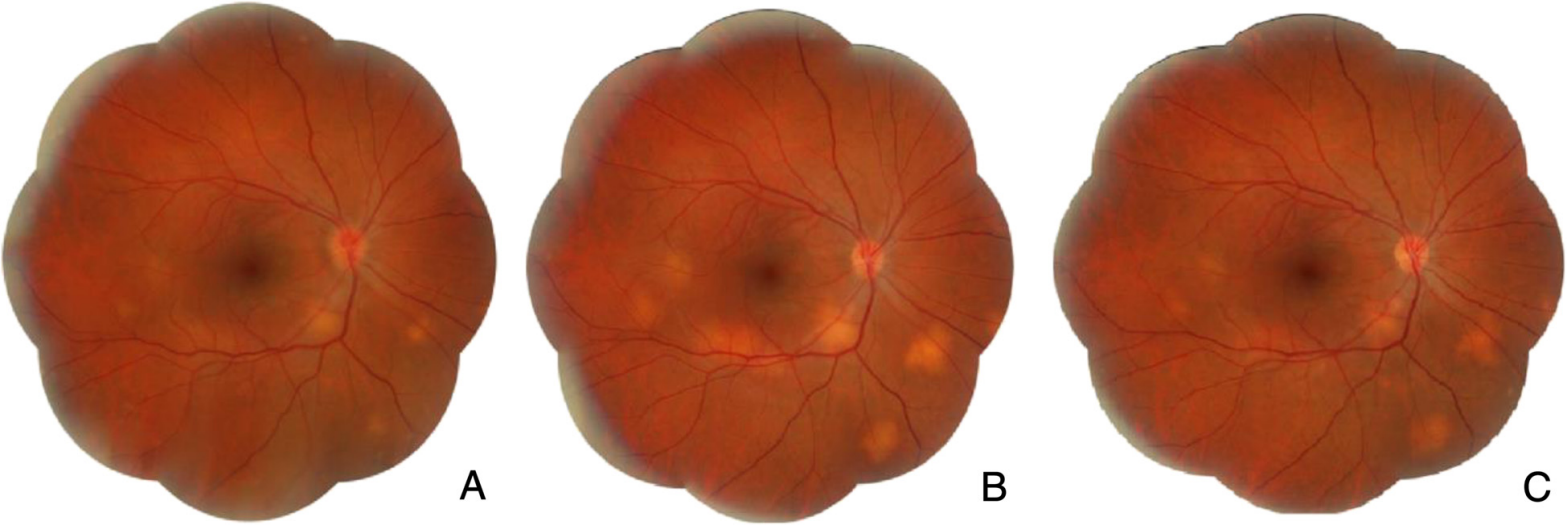
OD fundus. **a** Initial visit: optic disc hyperemia and edema and several yellowish subretinal lesions located in the posterior pole and lower midperiphery. **b** After 6-month therapy: decreased optic disc hyperemia and edema and increased choroidal granulomas. **c** After 18-month therapy: decreased choroidal granulomas

**Fig. 2:**
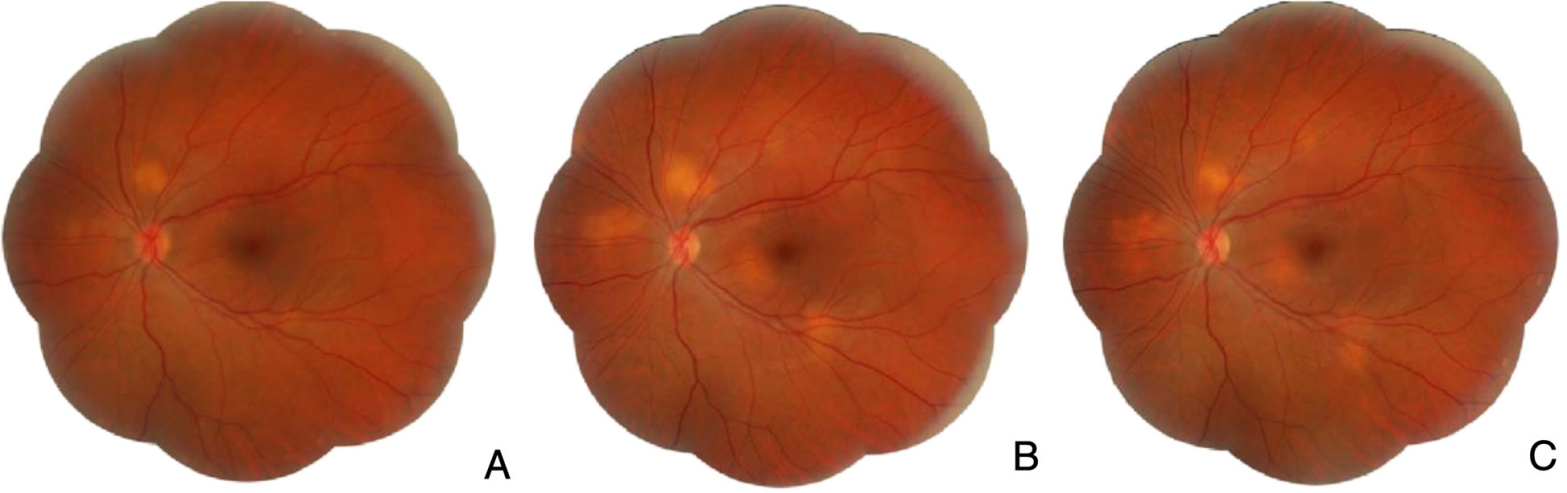
OS fundus. **a** Initial visit: optic disc hyperemia and edema and several yellowish subretinal lesions located in the posterior pole and lower midperiphery. **b** After 6-month therapy: decreased optic disc hyperemia and edema and increased choroidal granulomas. **c** After 18-month therapy: decreased choroidal granulomas

A 31 years-old caucasian female presented a 10-day history of decreased vision and pain in the OS. Examination showed visual acuity of 20/20 in OD and 20/25 in OS. Biomicroscopy revealed fine keratic precipitates, a mild reaction in the anterior chamber and in the vitreous cavity in both eyes and a small posterior synechiae in OS. Intraocular pressure was 12 mmHg in OD and 9 mmHg in OS. Fundoscopy disclosed mild swollen and hyperemic optic discs and some subretinal yellowish nodules of 1/3 to 1 disc diameter, partially delimited, located in the posterior pole and midperiphery in both eyes (Figs. 1a and 2a).

**Fig. 3:**
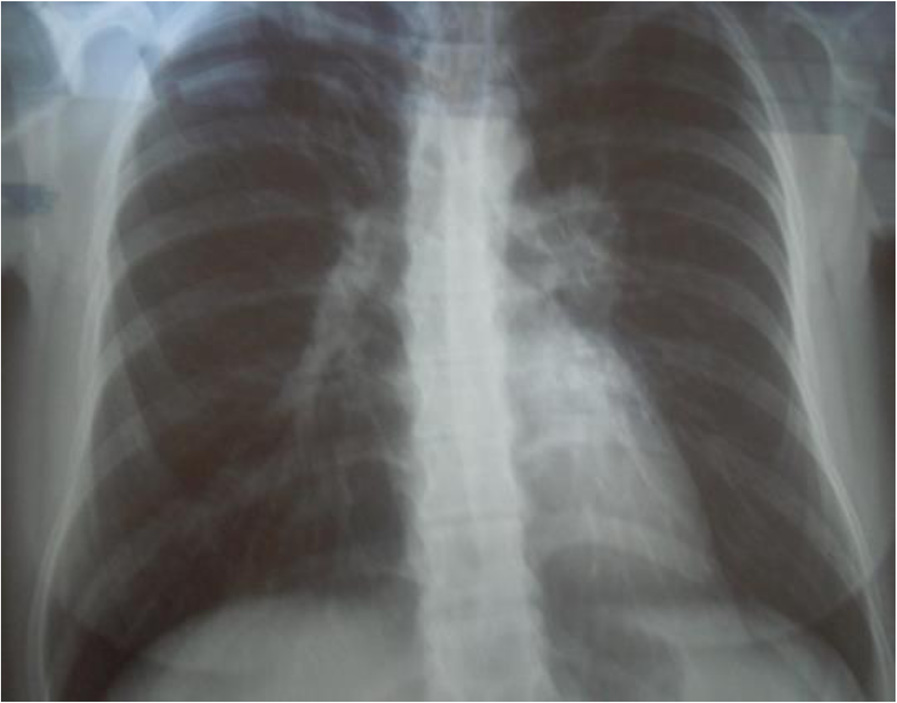
Chest X-ray. Chest X-ray showing a suspicion of hilar lymphadenopathy, especially on the left-side

**Fig. 4:**
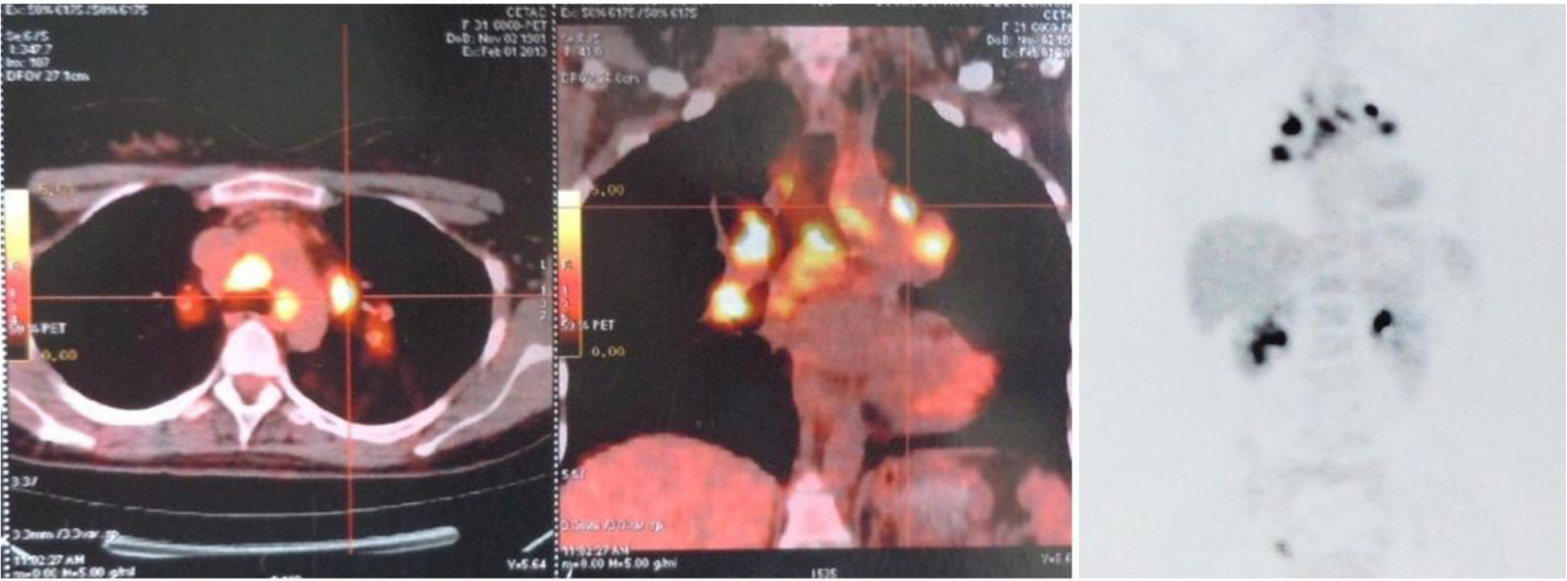
PET-Scan. PET-Scan showing increased 18 F-fluorodeoxyglucose uptake in multiple paratracheal and bilateral hilar lymph nodes

**Fig. 5:**
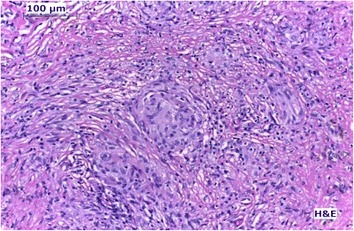
Histopathology. Histopathology of mediastinal lymph node, stained with hematoxylin-eosin, showing non-caseating granulomatous inflammation and multinucleated giant cells

Serologic tests for toxoplasmosis, syphilis and acquired immunodeficiency syndrome were negative as well as skin test for tuberculosis (purified protein derivative or PPD). Serum lysozyme was 14.0 mg/L (laboratory normal range 9,6 to 17,1 mg/L) and angiotensin-converting enzyme was 87.0 IU/L (laboratory normal range 35 to 90 IU/L). The Chest X-ray disclosed the suspicion of hilar lymphadenopathy, especially on the left-side (Fig. 3). The PET-scan showed increased 18 F-fluorodeoxyglucose uptake in the paratracheal and bilateral hilar lymph nodes, indicating high local cellular metabolism (Fig. 4). A mediastinal lymph node biopsy was performed afterwards, which depicted non-caseating granulomas with multinucleated giant cells and absence of acid-alcohol resistant bacilli (Fig. 5).

The patient was initially treated with oral prednisone 60 mg/day with gradual reduction. Six months after the treatment began the prednisone intake was 20 mg/day, when there was a decrease in papillitis and an increase in the choroidal granuloma size (Figs. 1b and 2b). At that visit a combination of methotrexate 20 mg/week and prednisone 60 mg/day was prescribed. During the following 12 months the prednisone daily dose was decreased 10 mg each visit. By the 18th month of treatment the intake of prednisone was 10 mg/day, leading to a gradual reduction in the choroidal granulomas’ size and thickness and stabilized uncorrected visual acuity of 20/20 in both eyes (Figs. 1c and 2c).

## Discussion

Ocular sarcoidosis is still rarely diagnosed in Brazil. In 2004, Gouveia et al. observed a prevalence of 2.29 % among of the causes of uveitis among all age groups in São Paulo (SP) [6].

The clinical suspicion of sarcoidosis requires a biopsy to confirm the diagnosis. When the disease causes only ocular manifestations, the diagnosis is more difficult and may be based on ophthalmologic evaluation and eventual findings of a systemic granulomatous inflammation, such as abnormal serologic tests and chest imaging [7]. Intraocular biopsy is usually not performed in these situations, due to its inherent risk of causing severe visual complications.

In the present case, ocular sarcoidosis was not accompanied by other clinical complaints. In addition, laboratory evaluation displayed normal values of serum lysozyme and angiotensin-converting enzyme. However, patients with active ocular sarcoidosis may be in systemic remission already, justifying a possible decrease in angiotensin-converting enzyme values [8]. Moreover, a suspected chest radiography image motivated additional workup and a definite diagnosis of sarcoidosis through mediastinal lymph node biopsy.

A weak response to steroid monotherapy was initially observed in this patient. The introduction of a non-steroid immunosuppressant drug (methotrexate) was associated with significant anatomic improvement in the following months. This fact drew attention because systemic steroids are the first line of therapy for sarcoidosis and provide faster and better outcomes even before the combination with other pharmacologic agents [9].

## Conclusion

Although sarcoidosis is a well-known disease, it rarely causes ocular abnormalities. The present case illustrates that ocular sarcoidosis may be unaccompanied by other clinical complaints or by elevated serum lysozyme and angiotensin-converting enzyme. Otherwise, complementary chest imaging and mediastinal lymph node biopsy were critical to make the diagnosis and start prompt therapy. This report raises the suggestion that an early introduction of a nonsteroidal immunosuppressive drug to systemic steroids may be beneficial for a rapid remission of the uveitis. Further studies with bigger number of cases are needed to draw a definite conclusion.

## Consent

Written informed consent was obtained from the patient for publication of this Case report and any accompanying images. A copy of the written consent is available for review by the Editor-in-Chief of this journal.

## Abbreviation

PET-scan: Positron emission tomography
